# Risk of Venous Thromboembolism after New Onset Heart Failure

**DOI:** 10.1038/s41598-019-53641-0

**Published:** 2019-11-22

**Authors:** Nathaniel R. Smilowitz, Qi Zhao, Li Wang, Sulena Shrestha, Onur Baser, Jeffrey S. Berger

**Affiliations:** 1 Leon H. Charney Division of Cardiology, Department of Medicine, New York University School of Medicine, New York, NY USA; 2 Division of Cardiology, Department of Medicine, Veterans Affairs New York Harbor Health Care System, New York, NY USA; 3 Janssen Pharmaceuticals, Beerse, Belgium; 4 STATinMED Research, Plano, TX USA; 5 MEF University, Istanbul, Turkey; 6 Division of Vascular Surgery, Department of Surgery, New York University School of Medicine, New York, NY USA

**Keywords:** Embolism, Epidemiology, Risk factors, Comorbidities

## Abstract

New-onset heart failure (HF) is associated with cardiovascular morbidity and mortality. It is uncertain to what extent HF confers an increased risk of venous thromboembolism (VTE). Adults ≥65 years old hospitalized with a new diagnosis of HF were identified from Medicare claims from 2007–2013. We identified the incidence, predictors and outcomes of VTE in HF. We compared VTE incidence during follow-up after HF hospitalization with a corresponding period 1-year prior to the HF diagnosis. Among 207,535 patients with a new HF diagnosis, the cumulative incidence of VTE was 1.4%, 2.5%, and 10.5% at 30 days, 1 year, and 5 years, respectively. The odds of VTE were greatest immediately after new-onset HF and steadily declined over time (OR 2.2 [95% CI 2.0–2.3], OR 1.5 [1.4–1.7], and OR 1.2 [1.2–1.3] at 0–30 days, 4–6 months, and 7–9 months, respectively). Over 26-month follow-up, patients with HF were at two-fold higher risk of VTE than patients without HF (adjusted HR 2.31 [2.18–2.45]). VTE during follow-up was associated with long-term mortality (adjusted HR 1.60, 95% CI 1.56–1.64). In conclusion, patients with HF are at increased risk of VTE early after a new HF diagnosis. VTE in patients with HF is associated with long-term mortality.

## Introduction

The prevalence of heart failure (HF) has been on the rise among older adults in the United States^1^. Heart failure is associated with substantial risk of both nonfatal and fatal major adverse cardiovascular events^1^. Nonfatal events, including myocardial infarction, stroke, recurrent HF, and venous thromboembolism (VTE), contribute to costs of care, impaired quality of life, and increased risk of all-cause mortality. Although VTE is common in hospitalized patients, it is uncertain to what extent a new diagnosis of HF contributes to VTE risk, with conflicting data from observational series and randomized trials^2^. Furthermore, among patients hospitalized for new-onset HF, the duration of time for which an excess risk of VTE persists after hospital discharge has not been characterized. These associations are important to develop prognostic models for VTE in HF, and to determine best practices regarding VTE surveillance and thromboprophylaxis after hospital discharge. Furthermore, clarification of the prognostic importance of VTE may impact the design of clinical trials in this high-risk population. Therefore, data are needed to rigorously assess the risk of VTE after hospitalization for new-onset HF. We used a random 5% sample of a national Medicare claims database to assess the magnitude and duration of increased VTE risk in a large population-based cohort with new diagnoses of HF. We also explored risk factors for VTE and the association between incident VTE and long-term mortality in patients with new onset HF.

## Methods

### Study population

Adults aged ≥65 years old with ≥1 inpatient claim for new onset HF from January 1^st^, 2007 through December 31^st^, 2013 were identified from a 5% random sample of a Medicare claims database (n = 356,450). Heart failure was defined by International Classification of Diseases, Ninth Revision, Clinical Modification (ICD-9) diagnosis codes 428.xx. The discharge date of the first inpatient claim for HF was defined as the index date for follow-up. Patients were eligible for study inclusion if they had continuous Medicare health plan enrollment with medical and pharmacy benefits for at least 1 year prior to the index date (n = 321,588). The one-year period prior to the diagnosis of HF was defined as the baseline period. To restrict the study to new onset HF, patients with diagnosis codes for HF during the 1-year baseline period were excluded from the analysis (n = 114,053).

### Study outcome

The primary study outcome was any acute VTE, defined by deep venous thrombosis or pulmonary embolism. To define these as acute cases of VTE, we required a 6-month period free from thromboembolism before a recurrent VTE event could be established^3^. We identified VTE using validated ICD-9 diagnosis-codes (415.1, 453.4, 453.8) that were previously shown to have a positive predictive value of 94% or more^4^. Patients were followed until death, the end of continuous Medicare enrollment, or the end date of the study period, whichever occurred earlier. All-cause death was recorded for each individual.

### Crossover-cohort analysis

To characterize the timing and duration of increased VTE risk associated with new onset HF, we performed a retrospective crossover-cohort analysis. In this study design each patient serves as his or her own control to reduce unmeasured confounding. We compared the incidence of VTE during the 30-day follow-up period after hospitalization with a new diagnosis of HF (the time period beginning on the index date) to the incidence of VTE in the first 30 days of the baseline period (the HF-free time period beginning 1 year prior to the first diagnosis of HF)^5^. Cumulative incidences of venous thromboembolism were also compared during time intervals of 2–3 months, 4–6 months and 7–9 months in baseline and follow up periods. Data from the final 3 months of the baseline period prior to the index HF admission were excluded from the crossover-cohort analysis due to the potential for the effects of contamination from undiagnosed decompensated HF. We used univariate logistic regression to calculate odds ratios for the incidence of VTE after a diagnosis of HF relative to the risk in the same patients during an equivalent time period 1 year earlier.

### Matched analysis

Patients with new onset HF were matched 1:1 by age, sex, and year of hospitalization to adults who had at least 1 inpatient claim during study period and did not develop HF. In order to identify a representative cohort, the index date for patients without HF was defined by a randomly selected inpatient claim. A pool of 664,858 patients without new onset HF met study eligibility criteria and were used for 1:1 matching without replacement. The risk of VTE in patients hospitalized with HF (compared with patients without HF) was determined in unadjusted analyses and in a Cox proportional hazards model, after adjusting for demographics and clinical covariates. Clinical covariates included obesity, hypertension, hyperlipidemia, diabetes mellitus, chronic renal disease, coronary artery disease, peripheral arterial disease, prior coronary artery bypass grafting (CABG), prior percutaneous coronary intervention (PCI), prior myocardial infarction, arrhythmias, ischemic stroke, transient ischemic attack, malignancy, anemia, pulmonary edema, anasarca, hepatic disease, thrombophilia, peptic ulcer disease, bleeding diathesis, major bleeding, pneumonia, COPD, depression, dementia, varicose veins, coagulation defect, rheumatoid arthritis, inflammatory bowel disease, alcohol abuse, and trauma. The use of antithrombotic therapy was not available from this Medicare dataset and therefore was not included as a covariate for adjustment.

### Statistical analysis

Continuous variables were displayed as means and standard deviations and were compared by student’s t-tests. Categorical variables were compared by chi square tests. Event rates and cumulative incidence estimates for VTE after HF were reported. Logistic regression analyses were performed to estimate odds of VTE associated with demographics and clinical characteristics in patients with HF. Time varying Cox proportional hazards models were generated to estimate the association between a first diagnosis of VTE and long-term mortality in patients with a new diagnosis of HF. Kaplan Meier curves were generated to illustrate long-term survival free from VTE in patients with and without new onset diagnoses of HF. All analyses were performed in SAS (SAS Institute, Carey, NC). All methods were carried out in accordance with relevant guidelines and regulations. The Medicare database used is commercially available and fully de-identified. Since the study did not involve interaction with human subjects or access to identifiable information, the study was not considered to be human subjects research by the New York University School of Medicine institutional review board (IRB) and therefore did not require IRB review.

## Results

A total of 207,535 adults ≥65 years of age met eligibility criteria with continuous health plan enrollment, ≥1 inpatient claim for HF during the study period, and without prior HF during the 1-year baseline period. Baseline characteristics of this cohort are shown in Supplemental Table 1.

**Figure 1:**
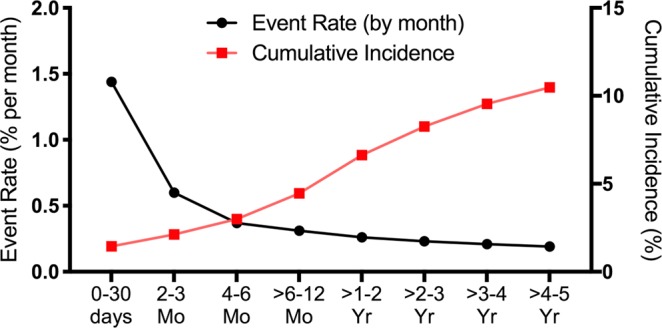
Monthly VTE event rate and cumulative incidence of VTE over time among patients with a new diagnosis of heart failure (n = 207,535).

Overall, 18,829 patients with HF developed a VTE during follow up (mean follow-up of 25.6 ± 24.1 months). Rates of VTE were highest in the first 30 days after a new diagnosis of HF and declined over time (Fig. 1). The cumulative incidence of VTE was 1.44% (1.39–1.49%) at 30 days after a new HF diagnosis, 4.45% (4.36–4.54%) at 1 year, and 10.48% (10.33–10.63%) at 5 years. The monthly VTE event rate was highest in the first 3-months after discharge (144 events per 10,000 new HF patients for the period of 0–30 days and 60 events per 10,000 new HF patients for the period of 2–3 months). The event rate per month steadily decreased after 3-months and plateaued at ≈20 events per 10,000 HF patients per month after year 2. Patients with prior VTE had consistently higher rates of acute VTE after a new diagnosis of HF than those without prior VTE (Supplemental Fig. 1, Supplemental Tables 1, 2).

The risks of VTE before and after the onset of HF in a crossover-cohort analysis are shown in Table 1. The risk of VTE was more than two-fold higher within 3 months of a HF diagnosis than during the same period 1-year prior (odds ratio of 2.16 [95% CI 2.02–2.30] and 2.08 [95% CI 1.94–2.23] for VTE within 30 days and 2–3 months, respectively). In the period of 4 to 6 months after hospital discharge for HF, there was 50% increased odds of VTE (OR 1.54 [95% CI 1.44–1.65]), as compared with the same period 1 year prior. For the period of 7 to 9 months, there was a modest, but still significantly increased risk of VTE, corresponding to an odds ratio of 1.24 (95% CI 1.15–1.33).

**Table 1:** Risk of VTE from a crossover-cohort analysis before and after the onset of heart failure (N = 207,535).

Baseline Period^†^			After HF Diagnosis			
Time Interval	No. at Risk	No. VTE during interval	No. at Risk	No. VTE during interval	Unadjusted OR (95% CI)	Absolute increase in VTE risk per 10,000 patients per month
0–30 days	207,535	1,384	207,535	2,985	2.16 (2.02–2.30)	77.1
2–3 Mo	206,151	1,184	174,559	2,084	2.08 (1.94–2.23)	31.0
4–6 Mo	204,967	1,460	158,758	1,741	1.54 (1.44–1.65)	12.8
7–9 Mo	203,507	1,658	144,194	1,456	1.24 (1.15–1.33)	6.5

^†^The baseline period begins 1 year prior to the date of the new inpatient heart failure diagnsois.

Among all 207,535 patients with new onset HF, independent predictors of VTE during follow up included prior VTE (OR 3.47, 95% CI 3.31–3.62), diabetes mellitus (OR 1.55, 95% CI 1.47–1.63), black race (OR 1.49, 95% CI 1.42–1.57), prior stroke (OR 1.89, 95% CI 1.74–2.06) or TIA (OR 1.72, 95% CI 1.58–1.87), hypertension (OR 1.49, 95% CI 1.41–1.59), and older age [75–84 year] (OR 1.41, 95% CI 1.33–1.49). Factors independently associated with VTE in patients with HF are shown in Supplemental Table 3.

Among patients hospitalized with new onset HF (n = 207,535), 107,586 patients died over long-term follow up. Early mortality rates were high, with 79 deaths per 1,000 patients in the first 30 days after hospitalization, and then declined over time (Supplemental Fig. 2). A first diagnosis of VTE during the follow-up period was associated with long-term mortality (HR 1.60, 95% CI 1.56–1.64) after multivariable adjustment for demographics and clinical covariates. Other associations between clinical covariates and long-term mortality in patients with new onset HF are shown in Supplemental Table 4.

### Matched cohort

**Figure 2:**
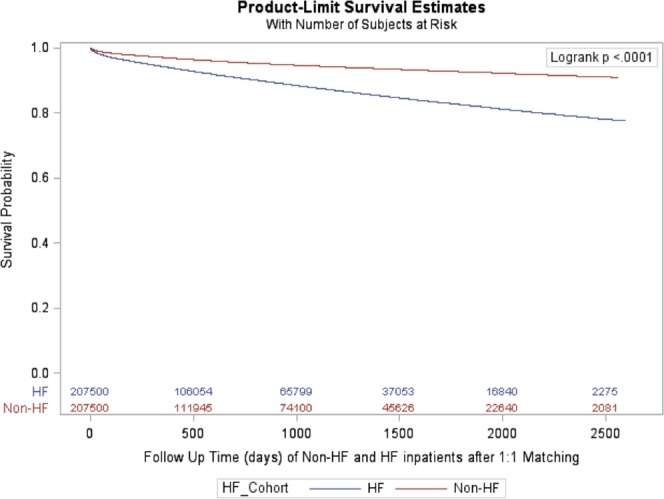
Kaplan meier survival free from VTE for patients with new-onset heart failure and matched patients without heart failure.

A total of 207,500 patients hospitalized with new onset HF were matched 1:1 to patients hospitalized without HF by age, sex, and year of index hospitalization. Baseline characteristics of patients with and without new onset HF are shown in Supplemental Table 5. Patients with new onset HF were at two-fold greater risk of incident VTE throughout the follow up period (mean follow-up 26 months) compared with patients without HF (Table 2). Kaplan Meier curves illustrating freedom from VTE in matched patients with and without HF are shown in Fig. 2. After adjustment for demographic and clinical covariates, a diagnosis of HF was independently associated with VTE (adjusted HR 2.31, 95% CI 2.18–2.45).

**Table 2:** Event rate and cumulative incidence of VTE during follow-up among matched patients with and without heart failure.

Time	Number at risk	Patients with Heart Failure (n = 207,500)			Patients without Heart Failure (n = 207,500)			
		No. with VTE	Event Rate %/month	Cumulative Incidence (%)	Number at risk	No. with VTE	Event Rate %/month	Cumulative Incidence (%)
0–30 days	207,500	2,985	1.44%	1.44% (1.39–1.49%)	207,500	1,544	0.74%	0.75% (0.71–0.79%)
2–3 Mo	174,542	2,084	0.60%	2.12% (2.06%, 2.19%)	175,201	1,124	0.32%	1.29% (1.25%, 1.34%)
4–6 Mo	158,747	1,741	0.37%	2.99% (2.92%, 3.07%)	160,124	867	0.18%	1.73% (1.68%, 1.79%)
6–12 Mo	144,186	2,715	0.32%	4.45% (4.36–4.54%)	146,153	1,282	0.15%	2.44% (2.37–2.51%)
1–2 Yr	120,623	3,749	0.26%	6.63% (6.52–6.75%)	124,872	1,681	0.11%	3.47% (3.38–3.55%)
2–3 Yr	86,429	2,403	0.23%	8.26% (8.13–8.39%)	93,090	911	0.08%	4.15% (4.06–4.25%)
3–4 Yr	60,748	1,549	0.21%	9.54% (9.40–9.68%)	68,363	578	0.07%	4.70% (4.60–4.81%)
4–5 Yr	40,549	902	0.19%	10.48% (10.33–10.63%)	48,456	358	0.06%	5.16% (5.05–5.28%)
>5 Yr	22,091	701	0.13%	11.25% (11.09–11.41%)	30,261	302	0.04%	5.69% (5.57–5.81%)

## Discussion

Heart failure affects more than 6.5 million Americans, with an increasing prevalence over the past decades^1^. A diagnosis of HF is associated with substantial short-term morbidity and mortality^6–8^. Most, although not all, studies have identified HF as an important risk factor for VTE^9–13^. Using administrative claims data from Medicare, we demonstrate that patients with HF are at increased risk for VTE. The greatest risk of VTE occurred within the first 3 months following a new HF diagnosis. Although the risk of VTE in HF persisted during the duration of the study, the magnitude of this risk declined over time and reached a plateau by 2 years. In a crossover-cohort analysis, a new diagnosis of HF conferred a significant increased risk of VTE that was most apparent in the first 3 months following hospital discharge. Finally, in a separate analysis of matched patients with and without HF, we identified a 2-fold higher incidence of VTE in patients with HF that remained significant over long-term follow up.

Our population-based data are largely consistent with prior observations that the risk of VTE is higher in patients with HF than in matched patients hospitalized without HF. Cross sectional data from the National Hospital Discharge Survey from 1979–2003 found that a diagnosis of HF was associated with a ≈50% increased relative risk of VTE, with the greatest excess risk in younger individuals, women, and patients of black race with HF^10^. However, this study was cross-sectional, did not report long-term risks of VTE after the initial diagnosis of HF, and matching and time-sequence analyses were not employed. A number of other analyses of small registries also report associations between HF and VTE^11^. In the SIRIUS case-control study, chronic HF was independently associated with a 3-fold increased risk of VTE^9^. Severe HF, defined by an elevated NT-proBNP was also associated with the risk of VTE in the MAGELLAN registry^12^. However, not all studies have reported consistent findings. In an analysis of 1,102 immobilized general medical inpatients enrolled in a multicenter randomized trial of low-molecular-weight heparin, no associations between HF and VTE were observed^13^. Furthermore, in the COMMANDER-HF trial of rivaroxaban 2.5 mg twice daily versus placebo in patients with HF, coronary artery disease and in sinus rhythm, only 0.6% of participants developed VTE over a median follow-up of 21.1 months. The low incidence of VTE in this cohort with HF is likely related to enrolling younger HF patients without atrial fibrillation. Furthermore, since the excess risk of VTE appears to be greatest early after a new diagnosis of HF, short trials in HF may not accurately reflect lifetime VTE risks in this high-risk population^14^. In addition to HF, other predictors of VTE include older age, reduced mobility, recent trauma, myocardial infarction, stroke, malignancy, and infectious disease^13,15,16^. We included these clinical comorbidities as covariates for risk adjustment in the current analysis to evaluate independent associations between heart failure VTE, and mortality.

In the present analysis, VTE in HF was independently associated with early mortality, consistent with prior analyses^17^. We also identified a long-term association between VTE and mortality that persisted over 5 years of follow-up. These data highlight the need for improved strategies for VTE diagnosis, prevention, and treatment in patients with HF. The 2012 American College of Physician clinical practice guidelines for VTE prophylaxis in hospitalized patients consider HF to be an important consideration in the decision to initiate VTE prophylaxis^18^. However, existing strategies to prevent VTE in patients with HF may be insufficient. In a randomized trial of hospitalized patients with medical illness, of whom 45% had HF, treatment with oral factor Xa inhibitor betrixiban up to 42 days was superior to 10 days of enoxaparin to reduce VTE. These data suggest that longer-term oral anticoagulation may provide additional opportunities to improve thromboprophylaxis in HF patients at risk for VTE.

### Limitations

There are important study limitations that must be acknowledged. First, the diagnosis of VTE was based on administrative coding data and approaches to VTE surveillance in this cohort were not standardized. Second, the present analysis was limited to a cohort of older adults with Medicare insurance coverage. Heart failure predominantly affects older individuals, and the results of this analysis of a Medicare database are broadly generalizable to most patients with heart failure, excluding young individuals and older adults without health insurance coverage. The strongest associations between VTE and HF have been reported in younger patients^10^, and thus this analysis may underestimate the impact of HF on the risk of VTE in this population. Third, data on baseline medications and the use of in-hospital thromboprophylaxis or outpatient anticoagulation were not available. However, in the modern era, patients routinely receive in-hospital thromboprophylaxis via mechanical or pharmacologic approaches to decrease the risk of VTE. Patients with HF are at higher risk for atrial fibrillation, which if appropriately treated with anticoagulation could bias the results of the current analysis towards the null hypothesis. Still, we observed a significant association between HF and new onset VTE. Fourth, measures of heart failure severity, including New York Heart Association (NYHA) Class and left ventricular ejection fraction were not available from this claims database. Finally, we cannot exclude the possibility of unmeasured confounders that were not included in the multivariable models performed in this analysis.

## Conclusions

Patients with HF are at increased risk of VTE, with the greatest risk within the first 3-months after a new inpatient diagnosis of HF. Venous thromboembolism in patients with HF was independently associated with long-term mortality. Further investigation is necessary to identify and treat HF patients at the highest risk of VTE.

## Supplementary information

Supplementary Materials
