# Supplementary material for: Risk of Venous Thromboembolism after New Onset Heart Failure
Source: Sci Rep. 2019 Nov 22;9:17415. doi: 10.1038/s41598-019-53641-0 (PMC6874686; doi:10.1038/s41598-019-53641-0)
Supplement: Supplementary file 1 — Supplementary Materials [file 41598_2019_53641_MOESM1_ESM.docx]

**Risk of Venous Thromboembolism after New Onset Heart Failure**

Nathaniel R. Smilowitz MD, MS ^1,2^, Qi Zhao, MD, MPH ^3^, Li Wang, MA, PhD, MBA^4^, Sulena Shrestha, MPH^4^, Onur Baser, MA, MS, PhD^5^, Jeffrey S. Berger MD, MS ^1,6*^

^1^ Leon H. Charney Division of Cardiology, Department of Medicine, New York University School of Medicine, New York, NY

^2^ Division of Cardiology, Department of Medicine, Veterans Affairs New York Harbor Health Care System, New York, NY

^3^ Janssen Pharmaceuticals

^4^ STATinMED Research, Plano, TX

^5^ The University of Michigan, Ann Arbor, MI

^6^ Division of Vascular Surgery, Department of Surgery, New York University School of Medicine, New York, NY

**SUPPLEMENTAL MATERIALS**

**Supplemental Table 1. Baseline Characteristics of heart failure inpatient population overall, and with and without prior VTE at baseline.**

|  | All HF Inpatients | |  | Without Baseline VTE | | With Baseline VTE | |  |
| --- | --- | --- | --- | --- | --- | --- | --- | --- |
|  | (N= 207,535) | |  | (N= 193,380) | | (N= 14,155) | |  |
|  | N/Mean | %/SD |  | N/Mean | %/SD | N/Mean | %/SD | p-value |
| Age (Mean) | 81.3 | 8.2 |  | 81.33 | 8.23 | 80.84 | 7.87 | <.0001 |
| Age Group |  |  |  |  |  |  |  |  |
| 65-74 | 48,757 | 23.49% |  | 45,374 | 23.46% | 3,383 | 23.90% | 0.2375 |
| 75-84 | 80,203 | 38.65% |  | 74,384 | 38.47% | 5,819 | 41.11% | <.0001 |
| 85+ | 78,575 | 37.86% |  | 73,622 | 38.07% | 4,953 | 34.99% | <.0001 |
| Female Sex | 129,710 | 62.50% |  | 120,557 | 62.34% | 9,153 | 64.66% | <.0001 |
| Race/Ethnicity |  |  |  |  |  |  |  |  |
| White | 181,225 | 87.32% |  | 169,193 | 87.49% | 12,032 | 85.00% | <.0001 |
| Black | 17,715 | 8.54% |  | 16,102 | 8.33% | 1,613 | 11.40% | <.0001 |
| Hispanic | 1,598 | 0.77% |  | 1,514 | 0.78% | 84 | 0.59% | 0.0128 |
| Asian | 2,407 | 1.16% |  | 2,291 | 1.18% | 116 | 0.82% | <.0001 |
| Native American | 3,377 | 1.63% |  | 3,132 | 1.62% | 245 | 1.73% | 0.3127 |
| Other | 913 | 0.44% |  | 869 | 0.45% | 44 | 0.31% | 0.0162 |
| Unknown | 300 | 0.14% |  | 279 | 0.14% | 21 | 0.15% | 0.9018 |
| US Geographic Region |  |  |  |  |  |  |  |  |
| Northeast | 42,039 | 20.26% |  | 39,240 | 20.29% | 2,799 | 19.77% | 0.139 |
| Midwest | 53,093 | 25.58% |  | 49,389 | 25.54% | 3,704 | 26.17% | 0.0986 |
| South | 83,671 | 40.32% |  | 77,972 | 40.32% | 5,699 | 40.26% | 0.8897 |
| West | 28,214 | 13.59% |  | 26,287 | 13.59% | 1,927 | 13.61% | 0.9462 |
| Other | 518 | 0.25% |  | 492 | 0.25% | 26 | 0.18% | 0.1035 |
| Comorbidity Indices |  |  |  |  |  |  |  |  |
| Charlson Comorbidity Index | 3.95 | 2.54 |  | 3.89 | 2.5 | 4.71 | 2.97 | <.0001 |
| CHA_2_DS_2_VASc Score |  |  |  |  |  |  |  |  |
| 0 | 0 | 0.00% |  | 0 | 0.00% | 0 | 0.00% | N/A |
| 1 | 300 | 0.14% |  | 294 | 0.15% | 6 | 0.04% | 0.0009 |
| 2 | 2,499 | 1.20% |  | 2,384 | 1.23% | 115 | 0.81% | <.0001 |
| ≥3 | 204,736 | 98.65% |  | 190,702 | 98.62% | 14,034 | 99.15% | <.0001 |
| Comorbid Conditions |  |  |  |  |  |  |  |  |
| Diabetes | 85,818 | 41.35% |  | 79,938 | 41.34% | 5,880 | 41.54% | 0.6362 |
| Hypertension | 171,245 | 82.51% |  | 159,229 | 82.34% | 12,016 | 84.89% | <.0001 |
| Peripheral Arterial Disease | 41,282 | 19.89% |  | 37,907 | 19.60% | 3,375 | 23.84% | <.0001 |
| Malignant Neoplasm | 49,614 | 23.91% |  | 45,148 | 23.35% | 4,466 | 31.55% | <.0001 |
| Myocardial Infarction | 46,639 | 22.47% |  | 43,627 | 22.56% | 3,012 | 21.28% | 0.0004 |
| Arterial Embolic Events | 1,735 | 0.84% |  | 1,432 | 0.74% | 303 | 2.14% | <.0001 |
| Dementia | 25,957 | 12.51% |  | 23,769 | 12.29% | 2,188 | 15.46% | <.0001 |
| Anemia | 98,144 | 47.29% |  | 89,867 | 46.47% | 8,277 | 58.47% | <.0001 |
| Pulmonary Edema | 1,235 | 0.60% |  | 1,122 | 0.58% | 113 | 0.80% | 0.0011 |
| Anasarca | 31,014 | 14.94% |  | 27,611 | 14.28% | 3,403 | 24.04% | <.0001 |
| Chronic Renal Insufficiency | 57,873 | 27.89% |  | 53,341 | 27.58% | 4,532 | 32.02% | <.0001 |
| Hepatic Disease | 10,046 | 4.84% |  | 9,016 | 4.66% | 1,030 | 7.28% | <.0001 |
| Thrombophilia | 4,898 | 2.36% |  | 4,055 | 2.10% | 843 | 5.96% | <.0001 |
| Peptic Ulcer | 1,806 | 0.87% |  | 1,614 | 0.83% | 192 | 1.36% | <.0001 |
| Bleeding Diathesis | 136 | 0.07% |  | 123 | 0.06% | 13 | 0.09% | 0.2051 |
| Chronic Obstructive Pulmonary Disease | 37,430 | 18.04% |  | 34,547 | 17.86% | 2,883 | 20.37% | <.0001 |
| Hyperlipidemia | 124,869 | 60.17% |  | 116,289 | 60.13% | 8,580 | 60.61% | 0.2605 |
| Depression | 8,251 | 3.98% |  | 7,496 | 3.88% | 755 | 5.33% | <.0001 |
| Obesity | 7,054 | 3.40% |  | 6,304 | 3.26% | 750 | 5.30% | <.0001 |
| Arrhythmia | 108,894 | 52.47% |  | 100,887 | 52.17% | 8,007 | 56.57% | <.0001 |
| Pneumonia | 58,493 | 28.18% |  | 53,382 | 27.60% | 5,111 | 36.11% | <.0001 |
| Varicose Veins | 5,547 | 2.67% |  | 4,931 | 2.55% | 616 | 4.35% | <.0001 |
| Coagulation Defect | 7,760 | 3.74% |  | 6,474 | 3.35% | 1,286 | 9.09% | <.0001 |
| Rheumatoid Arthritis | 9,089 | 4.38% |  | 8,264 | 4.27% | 825 | 5.83% | <.0001 |
| Inflammatory Bowel Disease | 1,949 | 0.94% |  | 1,740 | 0.90% | 209 | 1.48% | <.0001 |
| Alcohol Abuse | 1,582 | 0.76% |  | 1,455 | 0.75% | 127 | 0.90% | 0.0559 |
| Trauma | 62,825 | 30.27% |  | 57,276 | 29.62% | 5,549 | 39.20% | <.0001 |
| Established CAD | 119,797 | 57.72% |  | 112,084 | 57.96% | 7,713 | 54.49% | <.0001 |
| Prior Coronary Artery Bypass Grafting (CABG) | 5,745 | 2.77% |  | 5,372 | 2.78% | 373 | 2.64% | 0.3174 |
| Prior Percutaneous Coronary Intervention (PCI) | 9,953 | 4.80% |  | 9,493 | 4.91% | 460 | 3.25% | <.0001 |

**Supplemental Table 2. Event Rate and Cumulative Incidence of VTE during follow-up among inpatients with heart failure, with and without prior VTE**

|  |  | **Patients with HF without Prior VTE (n=193,380)** | | |  | **Patients with HF and with Prior VTE**  **(n=14,155)** | | | |
| --- | --- | --- | --- | --- | --- | --- | --- | --- | --- |
| **Time** | **Number at risk** | **No. with VTE** | **Event Rate %/month** | **Cumulative Incidence (%)** |  | **Number at risk** | **No. with VTE** | **Event Rate %/month** | **Cumulative Incidence (%)** |
| 0-30 days | 193,380 | 2,099 | 1.09% | 1.09% (1.04%-1.14%) |  | 14,155 | 886 | 6.26% | 6.26% (5.87%-6.67%) |
| 2-3 Mo | 164,031 | 1,960 | 0.60% | 2.10% (2.03%, 2.16%) |  | 10,524 | 124 | 0.59% | 7.15% (6.73%, 7.58%) |
| 4-6 Mo | 149,029 | 1,530 | 0.34% | 2.91% (2.83%, 2.99%) |  | 9,729 | 211 | 0.72% | 8.65% (8.19%, 9.12%) |
| >6-12 Mo | 135,679 | 2,084 | 0.26% | 4.09% (4.00%-4.18%) |  | 8,515 | 631 | 1.25% | 13.53% (12.97%-14.11%) |
| >1-2 Yr | 114,035 | 3,003 | 0.22% | 5.91% (5.80%-6.02%) |  | 6,591 | 746 | 0.94% | 19.78% (19.11%-20.47%) |
| >2-3 Yr | 82,000 | 2,037 | 0.21% | 7.30% (7.18%-7.42%) |  | 4,429 | 366 | 0.69% | 23.30% (22.57%-24.04%) |
| >3-4 Yr | 57,637 | 1,366 | 0.20% | 8.39% (8.26%-8.53%) |  | 3,111 | 183 | 0.49% | 25.47% (24.69%-26.25%) |
| >4-5 Yr | 38,519 | 819 | 0.18% | 9.19% (9.05%-9.33%) |  | 2,030 | 83 | 0.34% | 26.67% (25.87%-27.48%) |

**Supplemental Table 3. Parameters Independently Associated with follow-up VTE among inpatients with heart failure** **(N= 207,535).**

| Parameter | aOR (95% CI) | Wald Chi-Square | P-value |
| --- | --- | --- | --- |
| VTE prior to HF diagnosis | 3.47 (3.32 - 3.62) | 3072.4 | <.0001 |
| Age Group |  |  |  |
| 65-74 | Reference |  |  |
| 75-84 | 1.41 (1.33 - 1.49) | 138.2 | <.0001 |
| 85+ | 1.05 (0.99 - 1.11) | 2.1 | 0.14 |
| Female Sex | 1.2 (1.16 - 1.24) | 114.4 | <.0001 |
| Race/Ethnicity |  |  |  |
| White | Reference |  |  |
| Black | 1.49 (1.42 - 1.57) | 249.0 | <.0001 |
| Hispanic | 0.84 (0.69 - 1.02) | 3.1 | 0.08 |
| Asian | 0.75 (0.63 - 0.89) | 11.2 | 0.00 |
| Native American | 1.12 (0.99 - 1.26) | 3.2 | 0.07 |
| Other | 0.89 (0.69 - 1.13) | 1.0 | 0.33 |
| Unknown | 0.7 (0.43 - 1.14) | 2.1 | 0.15 |
| US Geographic Region |  |  |  |
| Northeast | Reference |  |  |
| Midwest | 0.96 (0.92 - 1.01) | 3.1 | 0.08 |
| South | 0.9 (0.87 - 0.94) | 22.6 | <.0001 |
| West | 0.95 (0.9 - 1) | 4.2 | 0.04 |
| Other | 0.54 (0.37 - 0.79) | 10.1 | 0.00 |
| Comorbidity Indices |  |  |  |
| Charlson Comorbidity Index | 0.98 (0.97 - 0.99) | 22.9 | <.0001 |
| CHADS2 Score | 0.7 (0.67 - 0.72) | 299.5 | <.0001 |
| Comorbid Conditions |  |  |  |
| Diabetes | 1.55 (1.47 - 1.63) | 278.3 | <.0001 |
| Hypertension | 1.49 (1.41 - 1.59) | 170.9 | <.0001 |
| Peripheral Arterial Disease | 1.1 (1.06 - 1.14) | 21.3 | <.0001 |
| Malignant Neoplasm | 1.1 (1.06 - 1.15) | 20.8 | <.0001 |
| Arterial Embolic Events | 1.47 (1.28 - 1.69) | 30.1 | <.0001 |
| Dementia | 0.66 (0.62 - 0.7) | 217.7 | <.0001 |
| Anemia | 0.98 (0.94 - 1.01) | 2.3 | 0.13 |
| Pulmonary Edema | 0.95 (0.77 - 1.16) | 0.3 | 0.58 |
| Anasarca | 1.1 (1.05 - 1.14) | 19.3 | <.0001 |
| Chronic Renal Insufficiency | 1.05 (1.01 - 1.09) | 4.8 | 0.03 |
| Hepatic Disease | 0.87 (0.81 - 0.93) | 14.7 | 0.00 |
| Thrombophilia | 1.1 (0.95 - 1.28) | 1.8 | 0.18 |
| Peptic Ulcer | 1.03 (0.88 - 1.22) | 0.2 | 0.69 |
| Bleeding Diathesis | 0.81 (0.44 - 1.49) | 0.4 | 0.51 |
| Chronic Obstructive Pulmonary Disease | 1 (0.95 - 1.04) | 0.1 | 0.81 |
| Hyperlipidemia | 1.05 (1.02 - 1.09) | 7.9 | 0.00 |
| Depression | 1.12 (1.04 - 1.21) | 9.2 | 0.00 |
| Obesity | 1.05 (0.97 - 1.13) | 1.2 | 0.27 |
| Arrhythmia | 0.96 (0.93 - 0.99) | 7.8 | 0.01 |
| Pneumonia | 0.87 (0.84 - 0.9) | 58.5 | <.0001 |
| Varicose Veins | 1.24 (1.13 - 1.35) | 23.7 | <.0001 |
| Coagulation Defect | 1.17 (1.04 - 1.32) | 7.0 | 0.01 |
| Rheumatoid Arthritis | 1.17 (1.09 - 1.25) | 19.4 | <.0001 |
| Inflammatory Bowel Disease | 1.1 (0.95 - 1.27) | 1.5 | 0.21 |
| Alcohol Abuse | 0.93 (0.77 - 1.11) | 0.7 | 0.41 |
| Trauma | 1.08 (1.04 - 1.11) | 18.4 | <.0001 |
| Established CAD | 1.05 (1.02 - 1.09) | 7.8 | 0.01 |
| Prior Coronary Artery Bypass Grafting (CABG) | 1.05 (0.96 - 1.15) | 1.1 | 0.30 |
| Prior Percutaneous Coronary Intervention (PCI) | 1 (0.93 - 1.08) | 0.0 | 0.96 |
| Ischemic Stroke | 1.89 (1.74 - 2.06) | 213.4 | <.0001 |
| Transient Ischemic Attack | 1.72 (1.58 - 1.87) | 164.2 | <.0001 |
| Myocardial Infarction | 0.88 (0.84 - 0.91) | 34.9 | <.0001 |
| Major Bleeding | 0.91 (0.84 - 0.98) | 6.6 | 0.01 |

**Supplemental Table 4. Association between first VTE and Long-Term Mortality in patients with heart failure** **(n=207,535).**

| **Parameter** | **aHR (95% CI)** | | **Wald Chi Square** | | **P-value** | |
| --- | --- | --- | --- | --- | --- | --- |
| VTE during Follow Up | 1.60 (1.56 - 1.63) | | 2127.388 | | <.0001 | |
| Age Group |  | |  | |  | |
| 65-74 | Reference | |  | |  | |
| 75-84 | 1.11 (1.08 - 1.14) | | 70.2513 | | <.0001 | |
| 85+ | 1.82 (1.78 - 1.87) | | 2279.1704 | | <.0001 | |
| Female Sex | 0.85 (0.84 - 0.87) | | 618.3628 | | <.0001 | |
| Race/Ethnicity |  | |  | |  | |
| White | Reference | |  | |  | |
| Black | 0.93 (0.92 - 0.96) | | 38.6916 | | <.0001 | |
| Hispanic | 0.95 (0.89 - 1.02) | | 1.7966 | | 0.1801 | |
| Asian | 0.92 (0.87 - 0.97) | | 9.0438 | | 0.0026 | |
| Native American | 0.88 (0.84 - 0.92) | | 31.2285 | | <.0001 | |
| Other | 0.92 (0.84 - 1) | | 3.6605 | | 0.0557 | |
| Unknown | 0.92 (0.79 - 1.08) | | 0.9562 | | 0.3281 | |
| US Geographic Region | |  | |  | |  |
| Northeast | Reference | |  | |  | |
| Midwest | 1.00 (0.98 - 1.01) | | 0.1348 | | 0.7135 | |
| South | 1.04 (1.02 - 1.06) | | 24.2761 | | <.0001 | |
| West | 1.00 (0.98 - 1.02) | | 0.1338 | | 0.7146 | |
| Other | 1.11 (0.99 - 1.25) | | 3.0814 | | 0.0792 | |
| Comorbidity Indices | |  | |  | |  |
| Charlson Comorbidity Index | 1.13 (1.13 - 1.14) | | 5422.1005 | | <.0001 | |
| CHADS2 Score | 1.26 (1.24 - 1.28) | | 706.5108 | | <.0001 | |
| Comorbid Conditions | |  | |  | |  |
| Diabetes | 0.70 (0.68 - 0.71) | | 1158.6185 | | <.0001 | |
| Hypertension | 0.67 (0.66 - 0.69) | | 1136.3366 | | <.0001 | |
| Peripheral Arterial Disease | 1.00 (0.98 - 1.01) | | 0.1406 | | 0.7077 | |
| Malignant Neoplasm | 0.91 (0.9 - 0.92) | | 147.181 | | <.0001 | |
| Arterial Embolic Events | 1.02 (0.96 - 1.08) | | 0.234 | | 0.6286 | |
| Dementia | 1.51 (1.49 - 1.54) | | 2429.1256 | | <.0001 | |
| Anemia | 1.16 (1.14 - 1.17) | | 545.8409 | | <.0001 | |
| Pulmonary Edema | 1.06 (0.98 - 1.14) | | 2.052 | | 0.152 | |
| Anasarca | 1.02 (1.00 - 1.04) | | 6.1749 | | 0.013 | |
| Chronic Renal Insufficiency | 0.97 (0.96 - 0.99) | | 13.4962 | | 0.0002 | |
| Hepatic Disease | 1.19 (1.15 - 1.22) | | 161.7253 | | <.0001 | |
| Thrombophilia | 0.97 (0.92 - 1.03) | | 0.8891 | | 0.3457 | |
| Peptic Ulcer | 0.99 (0.93 - 1.05) | | 0.1151 | | 0.7344 | |
| Bleeding Diathesis | 1.20 (0.98 - 1.48) | | 3.0885 | | 0.0788 | |
| Chronic Obstructive Pulmonary Disease | 1.19 (1.17 - 1.21) | | 527.1412 | | <.0001 | |
| Hyperlipidemia | 0.74 (0.74 - 0.75) | | 2293.5354 | | <.0001 | |
| Depression | 0.98 (0.95 - 1.01) | | 1.5958 | | 0.2065 | |
| Obesity | 0.88 (0.85 - 0.91) | | 43.2089 | | <.0001 | |
| Arrhythmia | 1.07 (1.06 - 1.08) | | 123.9984 | | <.0001 | |
| Pneumonia | 1.34 (1.32 - 1.35) | | 2035.6793 | | <.0001 | |
| Varicose Veins | 0.94 (0.91 - 0.98) | | 11.0151 | | 0.0009 | |
| Coagulation Defect | 1.10 (1.05 - 1.15) | | 16.4754 | | <.0001 | |
| Rheumatoid Arthritis | 0.92 (0.89 - 0.94) | | 38.7175 | | <.0001 | |
| Inflammatory Bowel Disease | 0.96 (0.90 - 1.02) | | 1.9956 | | 0.1578 | |
| Alcohol Abuse | 0.98 (0.91 - 1.05) | | 0.4852 | | 0.4861 | |
| Trauma | 1.06 (1.05 - 1.07) | | 84.5875 | | <.0001 | |
| Established CAD | 0.94 (0.92 - 0.95) | | 94.4216 | | <.0001 | |
| Prior Coronary Artery Bypass Grafting (CABG) | 0.52 (0.49 - 0.54) | | 718.766 | | <.0001 | |
| Prior Percutaneous Coronary Intervention (PCI) | 0.73 (0.71 - 0.76) | | 372.6533 | | <.0001 | |
| Ischemic Stroke | 0.63 (0.61 - 0.65) | | 688.4629 | | <.0001 | |
| Transient Ischemic Attack | 0.66 (0.64 - 0.68) | | 531.1088 | | <.0001 | |
| Myocardial Infarction | 1.14 (1.12 - 1.15) | | 238.1749 | | <.0001 | |
| Major Bleeding | 1.07 (1.05 - 1.10) | | 29.0456 | | <.0001 | |

**Supplemental Table 5. Baseline characteristics of heart failure and non-heart failure inpatients after 1:1 matching (total n=415,000)**

| Baseline Characteristics | Hospitalized with Heart Failure | | Hospitalized without Heart Failure | |  | |
| --- | --- | --- | --- | --- | --- | --- |
|  | (N=207,500) | | (N=207,500) | |  | |
|  | N/Mean | %/SD | N/Mean | %/SD | | p-value |
| Age (Mean) | 81.29 | 8.2 | 81.29 | 8.2 | | 1 |
| Age Group |  |  |  |  | |  |
| 65-74 | 48,757 | 23.50% | 48,757 | 23.50% | | 1 |
| 75-84 | 80,203 | 38.65% | 80,203 | 38.65% | | 1 |
| 85+ | 78,540 | 37.85% | 78,540 | 37.85% | | 1 |
| Female Sex | 129,690 | 62.50% | 129,690 | 62.50% | | 1 |
| Race/Ethnicity |  |  |  |  | |  |
| White | 181,199 | 87.32% | 183,997 | 88.67% | | <.0001 |
| Black | 17,709 | 8.53% | 14,512 | 6.99% | | <.0001 |
| Hispanic | 1,597 | 0.77% | 1,917 | 0.92% | | <.0001 |
| Asian | 2,407 | 1.16% | 2,693 | 1.30% | | <.0001 |
| Native American | 3,377 | 1.63% | 3,242 | 1.56% | | 0.0944 |
| Other | 911 | 0.44% | 792 | 0.38% | | 0.0039 |
| Unknown | 300 | 0.14% | 347 | 0.17% | | 0.0644 |
| US Geographic Region |  |  |  |  | |  |
| Northeast | 42,029 | 20.25% | 40,874 | 19.70% | | <.0001 |
| Midwest | 53,091 | 25.59% | 51,738 | 24.93% | | <.0001 |
| South | 83,654 | 40.32% | 79,534 | 38.33% | | <.0001 |
| West | 28,208 | 13.59% | 34,214 | 16.49% | | <.0001 |
| Other | 518 | 0.25% | 1,140 | 0.55% | | <.0001 |
| Comorbidity Indices |  |  |  |  | |  |
| Charlson Comorbidity Index | 3.95 | 2.54 | 1.82 | 2.26 | | <.0001 |
| CHA_2_DS_2_–VASc Score | 5.5 | 1.42 | 3.83 | 1.35 | | <.0001 |
| 0 | 0 | 0.00% | 0 | 0.00% | | N/A |
| 1 | 300 | 0.14% | 6,263 | 3.02% | | <.0001 |
| 2 | 2,499 | 1.20% | 25,452 | 12.27% | | <.0001 |
| ≥ 3 | 204,701 | 98.65% | 175,785 | 84.72% | | <.0001 |
| Comorbid Conditions |  |  |  |  | |  |
| Diabetes | 85,813 | 41.36% | 51,752 | 24.94% | | <.0001 |
| Hypertension | 171,224 | 82.52% | 142,033 | 68.45% | | <.0001 |
| Peripheral Arterial Disease | 41,277 | 19.89% | 21,107 | 10.17% | | <.0001 |
| Malignant Neoplasm | 49,607 | 23.91% | 50,756 | 24.46% | | <.0001 |
| Myocardial Infarction | 46,633 | 22.47% | 11,057 | 5.33% | | <.0001 |
| Arterial Embolic Events | 1,735 | 0.84% | 772 | 0.37% | | <.0001 |
| Dementia | 25,950 | 12.51% | 23,337 | 11.25% | | <.0001 |
| Anemia | 98,123 | 47.29% | 60,874 | 29.34% | | <.0001 |
| Pulmonary Edema | 1,235 | 0.60% | 150 | 0.07% | | <.0001 |
| Anasarca | 31,009 | 14.94% | 12,755 | 6.15% | | <.0001 |
| Chronic Renal Insufficiency | 57,856 | 27.88% | 20,431 | 9.85% | | <.0001 |
| Hepatic Disease | 10,046 | 4.84% | 6,462 | 3.11% | | <.0001 |
| Thrombophilia | 4,898 | 2.36% | 2,140 | 1.03% | | <.0001 |
| Peptic Ulcer | 1,805 | 0.87% | 1,155 | 0.56% | | <.0001 |
| Bleeding Diathesis | 136 | 0.07% | 89 | 0.04% | | 0.0017 |
| Chronic Obstructive Pulmonary Disease | 37,425 | 18.04% | 12,570 | 6.06% | | <.0001 |
| Hyperlipidemia | 124,858 | 60.17% | 102,087 | 49.20% | | <.0001 |
| Depression | 8,250 | 3.98% | 6,116 | 2.95% | | <.0001 |
| Obesity | 7,054 | 3.40% | 1,653 | 0.80% | | <.0001 |
| Arrhythmia | 108,875 | 52.47% | 42,821 | 20.64% | | <.0001 |
| Pneumonia | 58,476 | 28.18% | 19,282 | 9.29% | | <.0001 |
| Varicose Veins | 5,547 | 2.67% | 3,101 | 1.49% | | <.0001 |
| Coagulation Defect | 7,760 | 3.74% | 3,487 | 1.68% | | <.0001 |
| Rheumatoid Arthritis | 9,089 | 4.38% | 6,167 | 2.97% | | <.0001 |
| Inflammatory Bowel Disease | 1,949 | 0.94% | 1,529 | 0.74% | | <.0001 |
| Alcohol Abuse | 1,581 | 0.76% | 1,007 | 0.49% | | <.0001 |
| Trauma | 62,811 | 30.27% | 50,187 | 24.19% | | <.0001 |
| Established CAD | 119,782 | 57.73% | 51,188 | 24.67% | | <.0001 |
| Prior Coronary Artery Bypass Grafting | 5,745 | 2.77% | 1,256 | 0.61% | | <.0001 |
| Prior Percutaneous Coronary Intervention | 9,953 | 4.80% | 3,219 | 1.55% | | <.0001 |

**Supplemental Figure 1.** Kaplan Meier survival free from VTE among patients admitted with heart failure, with and without prior VTE (total n=207,535)


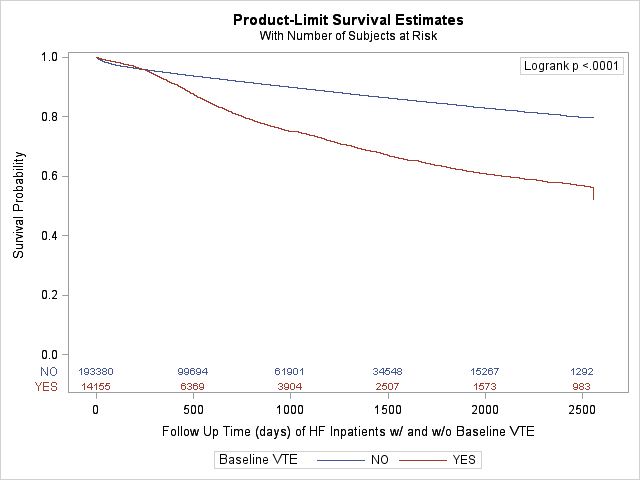


**Supplemental Figure 2.** Mortality rates among patients hospitalized with heart failure, over long-term follow up.

**
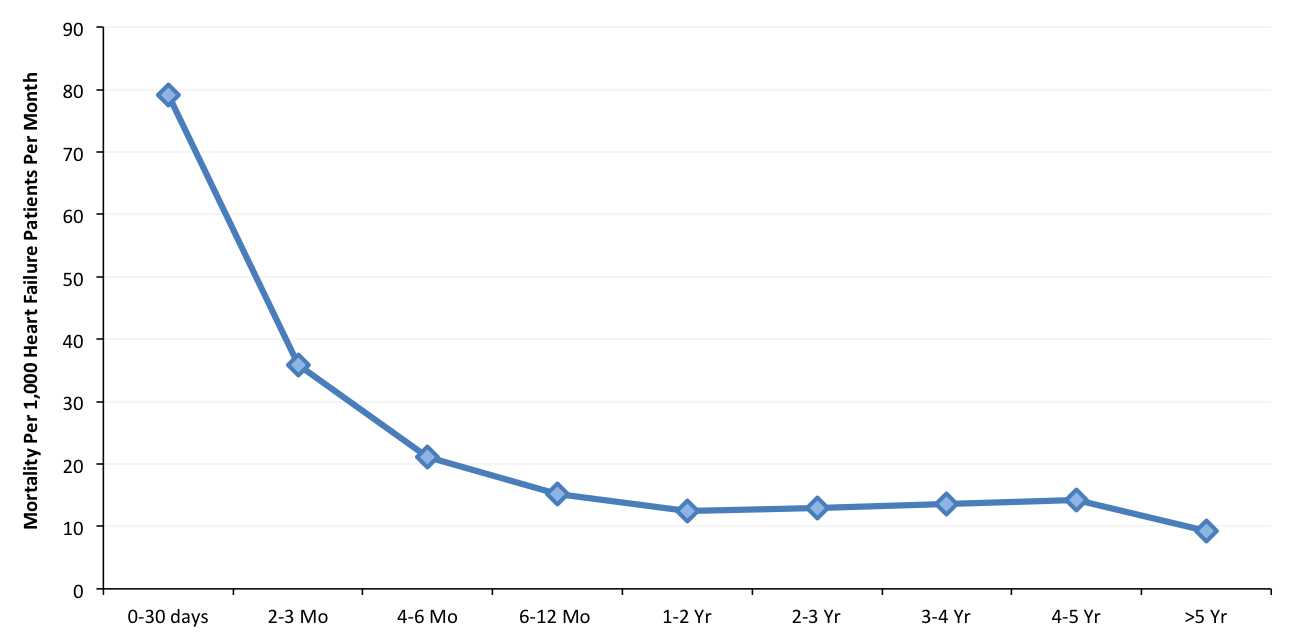
**
